# Pricing Interval European Option with the Principle of Maximum Entropy

**DOI:** 10.3390/e21080788

**Published:** 2019-08-13

**Authors:** Xiao Liu, Rongxi Zhou, Yahui Xiong, Yuexiang Yang

**Affiliations:** 1 School of Banking and Finance, University of International Business and Economics, Beijing 100029, China; 2 School of Management, China University of Mining and Technology (Beijing), Beijing 100083, China

**Keywords:** principle of maximum entropy, interval number, interval European option, option pricing

## Abstract

This paper develops the interval maximum entropy model for the interval European option valuation by estimating an underlying asset distribution. The refined solution for the model is obtained by the Lagrange multiplier. The particle swarm optimization algorithm is applied to calculate the density function of the underlying asset, which can be utilized to price the Shanghai Stock Exchange (SSE) 50 Exchange Trades Funds (ETF) option of China and the Boeing stock option of the United States. Results show that maximum entropy distribution provides precise estimations for the underlying asset of interval number situations. In this way, we can get the distribution of the underlying assets and apply it to the interval European option pricing in the financial market.

## 1. Introduction

Information entropy [1,2,3] has been widely used in financial engineering, especially where the maximum entropy method is employed within the field of option pricing [4,5,6]. It is important to note that the information utilized in the related models is limited to certain values, in such a way that the information from the financial market cannot be fully exploited. For example, the option price and the underlying asset price both have the highest and the lowest prices containing a large amount of information. It is therefore particularly necessary to study how to construct a maximum entropy model which can infer the distribution of the asset prices in the cases when the known option price has an uncertain value, such as being denoted by an interval number [7]. The probability distribution of the underlying asset can be deduced by using the interval price, and then the highest and lowest prices of the other relevant financial products can be predicted. If the lowest and highest prices of asset could be predicted based on the known information, then their corresponding risks can be controlled in advance. This would then provide a decision-making basis for asset pricing and risk management.

The principle of maximum entropy can be used in many fields. The maximum entropy solutions are shown to display a variety of behaviors if adequate dynamical information is inserted [8]. Elith et al. described the maximum entropy model from a statistical perspective [9]. Zambrano et al. proposed a thermodynamic model based on the maximum entropy principle, with dynamical prior information [10]. Hu et al. modeled the distribution changes of six typical Kobresia species in four periods by using the maximum entropy model [11]. Raney and Leopold used the maximum entropy model to estimate fen distribution throughout New York State [12]. In 1973, Cozzolino and Zahner first used the principle of maximum entropy to infer the probability distribution of future stock prices [13]. Based on the no-arbitrage principle, Bartiromo obtained the distribution of stock price fluctuations by maximizing information entropy [14].

In the field of option pricing, Jackwerth and Rubinstein used non-parametric methods to extract risk neutral probability distribution of the underlying asset [15]. Buchen and Kelly applied the principle of maximum entropy to estimate distribution of an underlying asset price [16]. Borwein et al. also revisited the maximum entropy principle and used partially finite convex programming to recover the probability distribution of an asset [17]. Similarly, Rompolis suggested a new method for implementing the principle of maximum entropy to retrieve the risk-neutral density of a future stock or of any other asset from the returns of European call and put prices [18]. Neri and Schneider also obtained the maximum entropy distribution of an asset from its call and digital option prices, while also offering detailed mathematical proof for its existence and exponential form [19]. Neri and Schneider also investigated the position of the Buchen-Kelly density within the family of entropy-maximizing densities, and observed that, within the market, all the densities matched European call option prices for a given maturity [20]. The entropy pricing theory established by Gulko showed that the distribution of future price changes was the most random, that was, the choice of price change obeyed the maximum entropy method [21,22]. Tapiero further emphasized the degree of uncertainty in statistical information with incomplete states, and modeled the cross-section of options bid-ask spreads with their strikes by maximizing the Kaniadakis entropy [23]. The above literature clearly shows that the entropy pricing theory can make full use of the known information and that the maximum entropy method has a certain degree of advantage when used in option pricing. The given information about a probability distribution (i.e., the distribution which maximizes entropy) is subject to constraints, but remains the least committal with respect to unknown or missing information and is for this reason also the least biased. The maximum entropy distribution is also a distribution which does not introduce correlations or additional structural details beyond those strictly required by the given constraints. 

This paper proposes an interval maximum entropy model to use the interval price obtained from the market as a representative of the limited information, while aiming at estimating the probability distribution of the asset price. In addition, the output of model can be applied to price the Shanghai Stock Exchange (SSE) 50 Exchange Trades Funds (ETF) option and the US Boeing stock option. For the linear optimization problem caused by the objective function and the constraint condition both being represented by interval numbers, Liu et al. proposed the concept of constraint satisfactory degree [24]. In this way, the issue can be transformed into a parametric linear programming problem with real coefficients.

The rest of this paper is organized as follows. Section 2 gives the definition of interval European option and introduces the interval maximum entropy model. The solution of model is obtained in this section. Section 3 shows the results of pricing interval European option from two empirical parts. Finally, Section 4 summarizes the paper.

## 2. The Interval Maximum Entropy Model

### 2.1. Interval European Option 

Interval European option is the European option where we selected data in the form of interval number, including the highest and lowest prices. Assume that, in the interval case, $D=[\underline{d},\bar{d}]$ is the interval option price corresponding to the strike price, $\underline{d}$ is the lowest price and $\bar{d}$ is the highest price. Suppose the asset prices are $\left[\underline{x},\bar{x}\right]$, similarly, the $\underline{x}$ represents the lowest price, while $\bar{x}$ represents the highest price.

Therefore, for the interval European option, the properties on the distribution can be expressed as an expectation, namely in the form of,

(1) $$\int_{0}^{\infty }p(x)C(x)dx=D,$$

where $C(x)=[\underline{c(x)},\bar{c(x)}]$ is the yield function that is known and it denotes the discounted expiry value of the option when the asset prices are $\left[\underline{x},\bar{x}\right]$. Note that $\underline{c(x)}$ is the lowest value, while $\bar{c(x)}$ is the highest value. Assume there is an interval European option which does not pay dividends. Under risk-neutral conditions, the yield function can be defined as a discounted function of the return on the maturity date T. Then, the call option yield function $C_{c}(x)$ and the put option yield function $C_{p}(x)$ are expressed as:

$$\begin{array}{cc} C_{c}(x) & =[\underline{c(x)},\bar{c(x)}]=D(T){\left([\underline{x},\bar{x}]-k\right)}^{+}=D(T)\max(0,[\underline{x},\bar{x}]-k)\\ & =D(T)\left[\max(0,\underline{x}-k),\max(0,\bar{x}-k)\right]\\ & =\left[D(T)\max(0,\underline{x}-k),D(T)\max(0,\bar{x}-k)\right] \end{array},$$

$$\begin{array}{cc} C_{p}(x) & =[\underline{c(x)},\bar{c(x)}]=D(T){\left(k-[\underline{x},\bar{x}]\right)}^{+}=D(T)\max(0,k-[\underline{x},\bar{x}])\\ & =D(T)\left[\max(0,k-\bar{x}),\max(0,k-\underline{x})\right]\\ & =\left[D(T)\max(0,k-\bar{x}),D(T)\max(0,k-\underline{x})\right] \end{array},$$

where k represents the strike price of option. The function D(T) denotes the non-stochastic present-value discount factor to time T and acts only as a multiplicative constant. For example, we choose $D(T)=e^{-rT}$ with a constant risk-free rate r.

### 2.2. Model Setting

Assume that market is arbitrage-free. Let X be a continuous random variable representing the price of some asset or security at some fixed expiration time T. In order to price the derivative, we need to estimate the probability density p(x) of X on 0<x<∞, using the information of a set of option interval prices from certain derivatives of X which expire at T. The entropy of the distribution p(x) is defined as

(2) $$S(p)=-\int_{0}^{\infty }p(x)\log p(x)dx.$$

Note that $\underset{p\to 0}{\lim}p\log p=0$, p(x)≥0 and $\int_{0}^{\infty }p(x)dx=1$.

Therefore, the interval maximum entropy model (IMEM) of interval European option with n strike prices can be established as follows:

(3) $$\begin{array}{l} \underset{p(x)}{\max}S(p)=-\int_{0}^{\infty }p(x)\log p(x)dx\\ s.t.\;\left\{ \begin{array}{l} \int_{0}^{\infty }p(x)dx=1\\ \int_{0}^{\infty }p(x)C_{i}(x)dx=D_{i}\\ p(x)\geq 0 \end{array}\right. \end{array}.$$

Here, $D_{i}=[\underline{d_{i}},\bar{d_{i}}]$ and $C_{i}(x)=[\underline{c_{i}(x)},\bar{c_{i}(x)}]$ (i=1,2…,n) are the interval option price and yield function corresponding to the ith strike price. The IMEM only relies on observable option prices, which can be expressed as expectations of known functions for the underlying asset price.

### 2.3. Solution of the Model

The IMEM is a constrained interval number optimization problem. When taking the option price into consideration, the objective function is a nonlinear function, while the constraint condition is an interval number function. The interval number constraint condition can be transformed into a deterministic constraint condition by using the constraint satisfactory degree of the constraint condition, which is proposed from literature [24].

For interval number $A=[\underline{a},\bar{a}]$, $B=[\underline{b},\bar{b}]$, let $len(A)=\bar{a}-\underline{a}$, $len(B)=\bar{b}-\underline{b}$. Thus $P(A\leq B)=\frac{\max(0,len(A)+len(B)-\max(0,\bar{a}-\underline{b}))}{len(A)+len(B)}$ represents the possibility degree of A≤B.

For each solution p(x) of the linear programming model with an interval number, let $\beta =P(\int_{0}^{\infty }p(x)C_{i}(x)dx\leq D_{i})$ represent the constraint satisfactory degree of p(x) to the ith constraint. Therefore, when the satisfactory degree is β (β∈[0,1]), the interval number constraint $\int_{0}^{\infty }p(x)C_{i}(x)dx\leq D_{i}$ can be transformed into a deterministic constraint, namely:

$$\left[\int_{0}^{\infty }\left(1-\beta )p(x\right)\underline{c_{i}(x)}+\beta p(x)\bar{c_{i}(x)}\right]dx\leq \beta \underline{d_{i}}+(1-\beta )\bar{d_{i}}.$$

Equation (3) can be transformed into a deterministic optimization problem in order to infer the probability density function (PDF) of the asset price and price the interval European option. First, we choose the satisfactory degree of 0 and 1 distinguishingly. Therefore, when β equals 0 and when it equals 1, Equation (3) is transformed into the following model:

(4) $$\begin{array}{l} \underset{p(x)}{\max}\;S_{0}(p)=-\int_{0}^{\infty }p(x)\log p(x)dx\\ s.t.\;\left\{ \begin{array}{l} \int_{0}^{\infty }p(x)dx=1\\ \int_{0}^{\infty }p(x)\underline{c_{i}}(x)dx=\bar{d_{i}}\\ p(x)\geq 0 \end{array}\right. \end{array}\begin{array}{l} \underset{p(x)}{\max}\;S_{1}(p)=-\int_{0}^{\infty }p(x)\log p(x)dx\\ s.t.\;\left\{ \begin{array}{l} \int_{0}^{\infty }p(x)dx=1\\ \int_{0}^{\infty }p(x)\bar{c_{i}(x)}dx=\underline{d_{i}}\\ p(x)\geq 0 \end{array}\right. \end{array}.$$

Equation (4) can further be simplified into the following unconstrained optimization problem [25]:

(5) $$S_{0\max}=\log u-\sum_{i=1}^{n}\lambda_{i}\bar{d_{i}},u=\int_{0}^{\infty }\exp(\sum_{i=1}^{n}\lambda_{i}\underline{c_{i}(x)})dx,$$

(6) $$S_{1\max}=\log u-\sum_{i=1}^{n}\lambda_{i}\underline{d_{i}},u=\int_{0}^{\infty }\exp(\sum_{i=1}^{n}\lambda_{i}\bar{c_{i}(x)})dx,$$

where $\lambda_{1}\ldots ,\lambda_{n}$ are the decision parameters. Second, for an arbitrary value of β, the model is:

(7) $$\begin{array}{l} \underset{p(x)}{\max}S(p)=-\int_{0}^{\infty }p(x)\log p(x)dx\\ s.t.\;\left\{ \begin{array}{l} \int_{0}^{\infty }p(x)dx=1\\ \left[\int_{0}^{\infty }\left(1-\beta )p(x\right)\underline{c_{i}(x)}+\beta p(x)\bar{c_{i}(x)}\right]dx=\beta \underline{d_{i}}+(1-\beta )\bar{d_{i}}\\ p(x)\geq 0 \end{array}\right. \end{array}.$$

The Lagrange multiplier method can be used to solve Equation (7). The corresponding Lagrange function is constructed according to its objective function and constraints, which can be expressed as follows:

$$H(p)=-\int_{0}^{\infty }p(x)\log p(x)dx+(1+\lambda_{0})\int_{0}^{\infty }p(x)dx+\sum_{i=1}^{n}\lambda_{i}\int_{0}^{\infty }\left(1-\beta )p(x\right)\underline{c_{i}(x)}+\beta p(x)\bar{c_{i}(x)}dx.$$

Here, $\lambda_{0},\lambda_{1}\ldots ,\lambda_{n}$ are all Lagrange multipliers.

H(p) will be maximized or minimized when its derivative, namely the first variation of H(p) with respect to the admissible variations of p(x), is zero. Additionally, the Hessian matrix is a diagonal matrix with the diagonal elements negative. Obviously, the Hessian matrix is negative definite and the function H(p) will achieve a maximum value. This leads to the following explicit representation of the IMEM:

$$\begin{array}{l} p(x)=\frac{1}{u}\exp(\sum_{i=1}^{n}((1-\beta )\lambda_{i}\underline{c_{i}(x)}+\beta \lambda_{i}\bar{c_{i}(x)})),\\ u=\int_{0}^{\infty }\exp(\sum_{i=1}^{n}((1-\beta )\lambda_{i}\underline{c_{i}(x)}+\beta \lambda_{i}\bar{c_{i}(x)}))dx=e^{-\lambda_{0}} \end{array}.$$

The original objective function can be simplified by $\lambda_{1}\ldots ,\lambda_{n}$, such that Equation (7) can be simplified into the following unconstrained optimization problem (NCOP):

(8) $$S_{\max}(\lambda_{i})=\log u-\sum_{i=1}^{n}\lambda_{i}\left(\beta \underline{d_{i}}+(1-\beta )\bar{d_{i}}\right).$$

Here, $u=\int_{0}^{\infty }\exp(\sum_{i=1}^{n}((1-\beta )\lambda_{i}\underline{c_{i}(x)}+\beta \lambda_{i}\bar{c_{i}(x)}))dx$, $\lambda_{1}\ldots ,\lambda_{n}$ are the decision variables.

The particle swarm optimization algorithm (developed by Kennedy and Eberhar in literature [26]) can be used to solve NCOP Equation (8). Equation (4) can therefore be regarded as a special case of general Equation (8) with the values of β=0 and β=1.

## 3. Empirical Analyses

Through the above process, the distribution of the underlying assets according to the interval option is obtained. Therefore, we can price the interval European option based on the known distribution.

When PDF of the future asset price is presented, the call interval European option price $D_{c}$ and the put interval European option price can be computed by:

$$\begin{array}{cc} D_{c} & =[\underline{d},\bar{d}]=\int_{0}^{\infty }p(x)C(x)dx\\ & =\int_{0}^{\infty }p(x)D(T){\left([\underline{x},\bar{x}]-k\right)}^{+}dx=\int_{0}^{\infty }p(x)D(T)\max(0,[\underline{x},\bar{x}]-k)dx \end{array}$$

$$\begin{array}{cc} D_{p} & =[\underline{d},\bar{d}]=\int_{0}^{\infty }p(x)C(x)dx\\ & =\int_{0}^{\infty }p(x)D(T){\left(k-[\underline{x},\bar{x}]\right)}^{+}dx=\int_{0}^{\infty }p(x)D(T)\max(0,k-[\underline{x},\bar{x}])dx \end{array}.$$

Hence, we obtain the distribution of the underlying assets according to the established model, and then can price the interval European option. This section consists of two parts, the first with the constraint satisfactory degree of 0 or 1. The second part considers the constraint satisfactory degree from 0 to 1, divided into 11 values. The data is selected from 50 ETF options and Boeing stock options. There is only one formal option in China, a 50 ETF option listed on the Shanghai stock exchange. In addition, according to Yahoo Finance, Boeing stock has the largest market cap in Industrials Services Sector. Therefore, we chose these two options. The former is a comprehensive index, which has only the systemic risk, no Idiosyncratic risk. The latter is a stock option, having both heterogeneous risk and systemic risk.

### 3.1. β Equal to 0 or 1

For the purpose of exploring the PDF of the asset price and interval option pricing when the constraint satisfactory degree is 0 and 1, respectively, we selected the China SSE 50 ETF call option [27,28]. This includes the highest and lowest prices, with the maturity date of 22 June and 28 September as established at 20 April 2016. The data is shown in Table 1. Accordingly, the risk-free rate $r_{f}$ is 2.2%. The current price of SSE 50ETF is 2.154.

Based on the information about the interval option prices for June, we calculated the PDF of the SSE 50 ETF price corresponding to the expiration date. We assumed that the PDF of the SSE 50 ETF price for September can be approximated. Selecting a constraint satisfactory degree of 0 and then of 1 results in two PDFs. Moreover, if the stock price is an interval number, the obtained option price is also an interval number. The price of the two obtained sets of intervals was compared with the actual price of the call option on 28 September.

**Figure 1:**
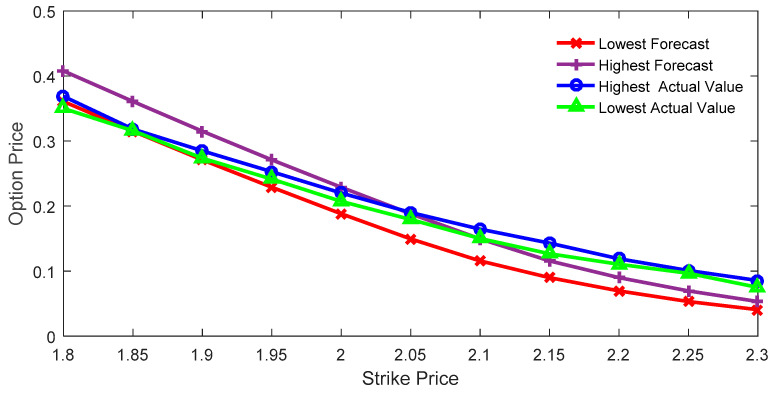
Interval price and actual option price with the constraint satisfactory degree of 0.

Figure 1 depicts the comparison between the estimated interval price and the actual interval price of the call option with the expiration date of 28 September. This was obtained with the IMEM for the case when the constraint satisfactory degree equals zero.

As can be seen from Figure 1, when the execution price is relatively smaller than the current price, the actual option price is within the predicted range. In other words, the lower bound of the predicted value is smaller than that of the actual value, and the upper bound of the predicted value is larger than that of the actual value. In contrast, when the execution price is relatively larger than current price, the actual option price is larger than the predicted price, that is, the upper bound of the predicted value is smaller than the lower bound of the actual value. The root mean square error (RMSE) is used to calculate the error between the market value and the estimated value. The RMSE of the highest price is 0.0265, while the RMSE of the lowest price is 0.0287.

**Figure 2:**
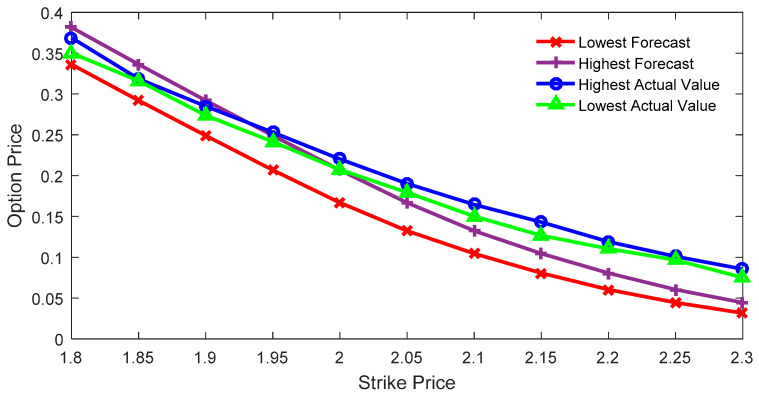
Interval price and actual option price with the constraint satisfactory degree of 1.

Figure 2 depicts the comparison between the estimated interval price and the actual interval price of the call option with the expiration date of September 28. This was obtained with the IMEM for the case when the constraint satisfactory degree equals one.

As can be seen from Figure 2, when the execution price is smaller, the actual option price is within the predicted range. In other words, the lower bound of the predicted value is smaller than that of the actual value, and the upper bound of the predicted value is larger than that of the actual value. When the execution price is relatively larger, the actual option price is larger than the predicted price, that is, the upper bound of the predicted value is smaller than the lower bound of the actual value. The RMSE of the highest price is 0.0293, while the RMSE of the lowest price is 0.0415. In Figure 1, it can easily be observed that the execution price at the point where the curve intersection changes has a value of 2.05. In Figure 2, the execution price at the point where the curve intersection changes has a value of 1.95.

### 3.2. β Belonging to [0, 1]

#### 3.2.1. China SSE 50 ETF Option Pricing

In order to examine the effect of the constraint satisfactory degree on the PDF of the underlying asset price and on the option pricing problem, we selected the call and put options of the China SSE 50 ETF. We included the highest and the lowest prices, with the maturity date of 28 March as established at the 20 November 2017 time point. The data is shown in Table 2. The quantity of strike price is 13. In this case, the $r_{f}$ is 4.01%. The current price of SSE 50ETF is 2.998.

We calculated the PDF of the SSE 50 ETF price corresponding to the expiration date based on the information about the interval call option prices for March. The constraint satisfactory degrees used were 0, 0.1, 0.2, 0.3, 0.4, 0.5, 0.6, 0.7, 0.8, 0.9, and 1, thus obtaining 11 corresponding PDFs. When the stock price is an interval number, then the obtained option price is also represented by an interval number. The PDF of the SSE 50 ETF price corresponding to the put option for the same maturity date remained consistent. As a result, the obtained interval prices were compared with the actual interval price of the put option with the same maturity date.

**Figure 3:**
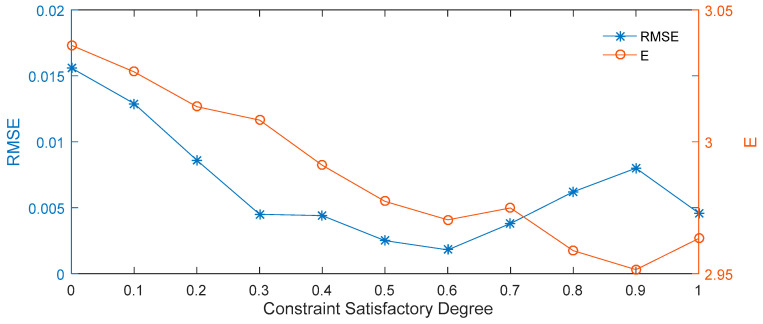
RMSE and expected prices for different constraint satisfactory degrees.

It can be seen from Table 3 and Figure 3 that the constraint satisfactory degree of β = 0.6 performs better for the lowest price prediction of the interval option price, and that the RMSE is the lowest for in this case. The observed trend is that the predicted expected price for the 50 ETF at the maturity date decreases with each increase in β. The highest expected price is obtained when β = 0, while the lowest expected price is obtained when β = 0.9.

**Figure 4:**
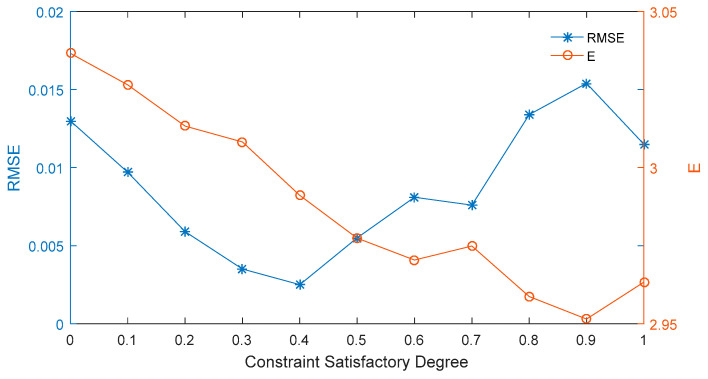
Root mean square error (RMSE) and expected prices at different constraint satisfactory degrees.

The results presented in Table 4 and Figure 4 demonstrate that when β = 0.4, then the forecast for the highest price of the interval option price is the best; at the same time, the RMSE is the lowest for in this case. Therefore, our comprehensive comparison of the forecasting effect on the interval price shows that the smallest RMSE value is obtained at the constraint satisfactory degree of 0.4. 

#### 3.2.2. US Boeing Stock Option Pricing

In order to examine the effect of the constraint satisfactory degree β on the PDF of the underlying asset price and on the option pricing problem, we selected the call and put options of the US Boeing stock. This includes the highest and the lowest prices, with the maturity date of 15 June as established at the 31 May 2018 time point. The data is shown in Table 5. The number of strike price is 10. The current price of Boeing stock is 3.5809. All price data was divided by 100. Accordingly, the $r_{f}$ is 1.83%.

We calculated the PDF of the Boeing stock price corresponding to the expiration date based on the information about the interval call option prices for June. The constraint satisfactory degrees used were 0, 0.1, 0.2, 0.3, 0.4, 0.5, 0.6, 0.7, 0.8, 0.9, and 1; therefore, we obtained 11 corresponding PDFs. For the case in which the stock price is an interval number, the option price was also obtained in the form of an interval number. The PDF of the Boeing stock price corresponding to the put option with the same maturity date remained consistent. The obtained interval prices were then compared to the actual interval price of the put option with the same maturity date.

**Figure 5:**
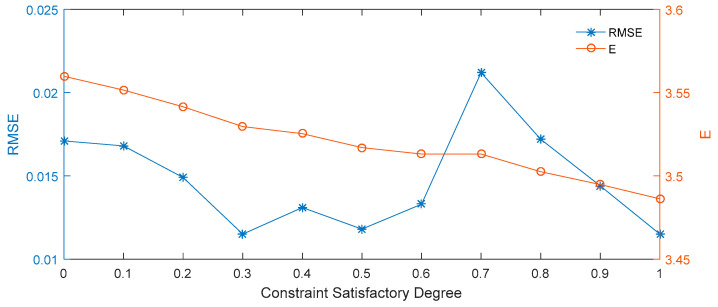
RMSE and expected prices at different constraint satisfactory degrees.

In Table 6, all price data has been divided by 100. From Table 6 and Figure 5, it can be seen that a value of β = 0.3 is better, as it leads to the lowest value of the interval option price and to the lowest RMSE value. The expected price of the Boeing stock at the expiration date roughly decreases with each increase in β. The highest expected price is obtained when β = 0, while the lowest expected price is obtained when β = 1.

**Figure 6:**
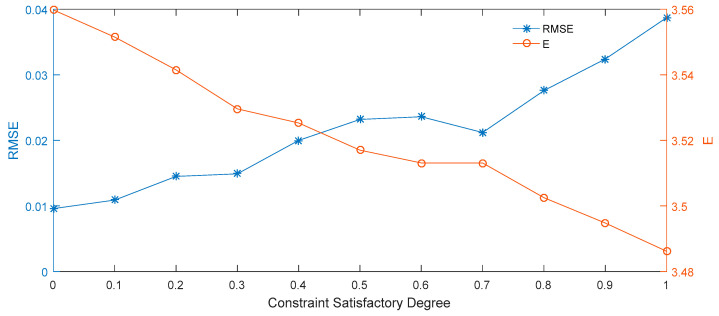
RMSE and expected prices at different constraint satisfactory degrees.

Once again, the price data in Table 4 has been divided by 100. The results in Table 7 and Figure 6 demonstrate that, when β = 0, then the forecast for the highest value of the interval option price is the most favorable, while also having the lowest RMSE value. Comprehensively comparing the forecasting effect observed on the interval price of the lowest and highest leads to obtaining the smallest RMSE when the constraint satisfactory degree is 0.3. 

In this part, the application of the model is designed. It is found that the distribution estimation of the underlying asset and the prediction effect of the interval option price are very good for options that exclude heterogeneity risk and options with heterogeneity risk, no matter whether the β is only 0 or 1, or the β is from 0 to 1. For different sample sets, the optimal constraint satisfactory degree (considering root mean square error of price prediction and the expectation of the underlying asset price) is different, which is influenced by the selected sample data. The best value in the Chinese data is 0.4, and the best value of US data is 0.3. β represents the weight of the lowest option price information when solving the probability density function. The smaller β means the smaller the weight of the lowest option price information and the greater the weight of the highest price information. The optimal value of β reflects the comprehensive utilization of highest price and lowest price information. The two optimal β values show that the highest and the lowest price information both have an effect on forecasting.

## 4. Conclusions

Considering that both the stock price and the option price are interval numbers, we developed an interval maximum entropy model with the constraint of the option price to infer the distribution of interval asset. Essentially, this represents an interval number programming problem. Based on the fuzzy constraint satisfactory method, we show that the interval number optimization problem can be reduced to a deterministic parameter optimization problem by setting the degree of constraint satisfactory. The Lagrange multiplier method is used to simplify the unconstrained programming problem in order to get an analytical solution. Using the particle swarm optimization algorithm to calculate the distribution, the empirical results of the SSE 50 ETF options of China and the US Boeing stock options are obtained. Our research shows that the interval maximum entropy model is suitable for obtain the distribution and the pricing of interval European options. 

Our method can provide a basis for investors to reflect whether the market pricing is reasonable, and help them to make options portfolios. The model also can give some market signals and early warning of pricing bias to companies issuing options. Future research should investigate the uses of this method in the effective risk management of options.

## Figures and Tables

**Table 1:** The highest and lowest prices for June and September.

50ETF June Call Option						50ETF September Call Option		
Strike Price	Highest	Lowest	Strike Price	Highest	Lowest	Strike Price	Highest	Lowest
1.80	0.3627	0.3400	2.25	0.0530	0.0436	1.80	0.3688	0.3502
1.85	0.3150	0.2928	2.30	0.0390	0.0321	1.85	0.3184	0.3160
1.90	0.2683	0.2492	2.35	0.0291	0.0241	1.90	0.2850	0.2737
1.95	0.2271	0.2066	2.40	0.0207	0.0162	1.95	0.2529	0.2415
2.00	0.1886	0.1684	2.45	0.0146	0.0115	2.00	0.2202	0.2072
2.05	0.1525	0.1329	2.50	0.0108	0.0085	2.05	0.1902	0.1793
2.10	0.1193	0.1061	2.55	0.0076	0.0061	2.10	0.1645	0.1501
2.15	0.0936	0.0801	2.60	0.0054	0.0045	2.15	0.1430	0.1266
2.20	0.0708	0.0605	2.65	0.0045	0.0036	2.20	0.1190	0.1105
						2.25	0.1010	0.0966
						2.30	0.0855	0.0750

**Table 2:** The highest and lowest price of call option and put option.

Strike Price		2.50	2.55	2.60	2.65	2.70	2.75	2.80	2.85	2.90	2.95	3.00	3.10	3.20
Call option	Highest	0.5309	0.4822	0.4350	0.3870	0.3425	0.2962	0.2520	0.2107	0.1740	0.1410	0.1122	0.0685	0.0400
	Lowest	0.4927	0.4455	0.3988	0.3502	0.3067	0.2624	0.1900	0.1765	0.1405	0.1100	0.0842	0.0471	0.0262
Put option	Highest	0.0062	0.0070	0.0088	0.0112	0.0143	0.0189	0.0254	0.0349	0.0481	0.0669	0.0901	0.1522	0.2287
	Lowest	0.0047	0.0051	0.0068	0.0086	0.0104	0.0144	0.0200	0.0279	0.0401	0.0550	0.0750	0.1308	0.2031

**Table 3:** The lowest forecast price of options under different constraint satisfactory degrees.

	β	0.0	0.1	0.2	0.3	0.4	0.5	0.6	0.7	0.8	0.9	1.0
*K*												
2.50		0.0032	0.0034	0.0060	0.0038	0.0042	0.0058	0.0042	0.0071	0.0052	0.0061	0.0054
2.55		0.0042	0.0044	0.0079	0.0050	0.0055	0.0075	0.0055	0.0093	0.0068	0.0080	0.0070
2.60		0.0053	0.0057	0.0099	0.0064	0.0070	0.0095	0.0070	0.0116	0.0087	0.0102	0.0088
2.65		0.0069	0.0074	0.0122	0.0083	0.0090	0.0117	0.0092	0.0141	0.0111	0.0131	0.0108
2.70		0.0090	0.0099	0.0148	0.0111	0.0117	0.0143	0.0121	0.0170	0.0145	0.0167	0.0134
2.75		0.0121	0.0131	0.0180	0.0151	0.0152	0.0175	0.0155	0.0202	0.0189	0.0209	0.0165
2.80		0.0159	0.0169	0.0219	0.0202	0.0195	0.0213	0.0198	0.0241	0.0247	0.0258	0.0207
2.85		0.0211	0.0221	0.0275	0.0269	0.0258	0.0270	0.0268	0.0304	0.0326	0.0334	0.0284
2.90		0.0295	0.0308	0.0367	0.0370	0.0364	0.0372	0.0385	0.0411	0.0434	0.0455	0.0405
2.95		0.0412	0.0432	0.0497	0.0518	0.0515	0.0530	0.0550	0.0560	0.0599	0.0627	0.0585
3.00		0.0566	0.0596	0.0673	0.0723	0.0706	0.0736	0.0769	0.0754	0.0840	0.0849	0.0813
3.10		0.1044	0.1094	0.1176	0.1256	0.1244	0.1301	0.1356	0.1321	0.1436	0.1453	0.1414
3.20		0.1612	0.1686	0.1782	0.1889	0.1905	0.1993	0.2054	0.2015	0.2145	0.2189	0.2120
RMSE		0.0156	0.0129	0.0086	0.0045	0.0044	0.0025	0.0018	0.0038	0.0062	0.0080	0.0046
E		3.0366	3.0265	3.0133	3.0082	2.9911	2.9774	2.9704	2.9749	2.9587	2.9515	2.9633

**Table 4:** The highest forecast price of options under different constraint satisfactory degrees.

	β	0.0	0.1	0.2	0.3	0.4	0.5	0.6	0.7	0.8	0.9	1.0
*K*												
2.50		0.0042	0.0044	0.0079	0.0050	0.0055	0.0075	0.0055	0.0093	0.0068	0.0080	0.0070
2.55		0.0053	0.0057	0.0099	0.0064	0.0070	0.0095	0.0070	0.0116	0.0087	0.0102	0.0088
2.60		0.0069	0.0074	0.0122	0.0083	0.0090	0.0117	0.0092	0.0141	0.0111	0.0131	0.0108
2.65		0.0090	0.0099	0.0148	0.0111	0.0117	0.0143	0.0121	0.0170	0.0145	0.0167	0.0134
2.70		0.0121	0.0131	0.0180	0.0151	0.0152	0.0175	0.0155	0.0202	0.0189	0.0209	0.0165
2.75		0.0159	0.0169	0.0219	0.0202	0.0195	0.0213	0.0198	0.0241	0.0247	0.0258	0.0207
2.80		0.0211	0.0221	0.0275	0.0269	0.0258	0.0270	0.0268	0.0304	0.0326	0.0334	0.0284
2.85		0.0295	0.0308	0.0367	0.0370	0.0364	0.0372	0.0385	0.0411	0.0434	0.0455	0.0405
2.90		0.0412	0.0432	0.0497	0.0518	0.0515	0.0530	0.0550	0.0560	0.0599	0.0627	0.0585
2.95		0.0566	0.0596	0.0673	0.0723	0.0706	0.0736	0.0769	0.0754	0.0840	0.0849	0.0813
3.00		0.0783	0.0821	0.0901	0.0972	0.0950	0.0994	0.1042	0.1012	0.1122	0.1127	0.1094
3.10		0.1324	0.1386	0.1474	0.1564	0.1566	0.1638	0.1697	0.1659	0.1780	0.1812	0.1760
3.20		0.1909	0.1993	0.2099	0.2226	0.2255	0.2360	0.2424	0.2382	0.2522	0.2578	0.2490
RMSE		0.0130	0.0097	0.0059	0.0035	0.0025	0.0055	0.0081	0.0076	0.0134	0.0154	0.0115
E		3.0366	3.0265	3.0133	3.0082	2.9911	2.9774	2.9704	2.9749	2.9587	2.9515	2.9633

**Table 5:** The highest and lowest price of call option and put option.

Strike Price		3.425	3.450	3.500	3.550	3.575	3.600	3.625	3.650	3.675	3.700
Call option	Highest	0.1720	0.1615	0.1241	0.0900	0.0720	0.0610	0.0490	0.0395	0.0305	0.0231
	Lowest	0.1615	0.1351	0.0991	0.0697	0.0575	0.0470	0.0385	0.0296	0.0225	0.0165
Put option	Highest	0.0261	0.0316	0.0463	0.0700	0.0780	0.0925	0.1075	0.1195	0.1335	0.1606
	Lowest	0.0170	0.0205	0.0315	0.0479	0.0585	0.0690	0.0855	0.0985	0.1203	0.1323

**Table 6:** The lowest forecast price of options under different constraint satisfactory degrees.

	β	0.0	0.1	0.2	0.3	0.4	0.5	0.6	0.7	0.8	0.9	1.0
*K*												
3.425		0.0152	0.0150	0.0157	0.0173	0.0157	0.0147	0.0117	0.0010	0.0010	0.0010	0.0011
3.450		0.0197	0.0194	0.0203	0.0224	0.0204	0.0191	0.0152	0.0033	0.0033	0.0035	0.0038
3.500		0.0305	0.0301	0.0313	0.0343	0.0311	0.0291	0.0241	0.0126	0.0130	0.0139	0.0153
3.550		0.0436	0.0431	0.0448	0.0485	0.0442	0.0423	0.0372	0.0281	0.0294	0.0314	0.0345
3.575		0.0510	0.0505	0.0525	0.0565	0.0520	0.0506	0.0462	0.0386	0.0404	0.0432	0.0472
3.600		0.0591	0.0587	0.0608	0.0651	0.0607	0.0601	0.0569	0.0507	0.0534	0.0570	0.0616
3.625		0.0679	0.0678	0.0700	0.0746	0.0706	0.0713	0.0695	0.0642	0.0679	0.0722	0.0775
3.650		0.0777	0.0779	0.0804	0.0851	0.0820	0.0840	0.0834	0.0787	0.0834	0.0882	0.0942
3.675		0.0885	0.0893	0.0921	0.0971	0.0953	0.0983	0.0986	0.0944	0.1001	0.1054	0.1118
3.700		0.1006	0.1021	0.1054	0.1108	0.1106	0.1144	0.1151	0.1114	0.1182	0.1236	0.1307
RMSE		0.0171	0.0168	0.0149	0.0115	0.0131	0.0118	0.0133	0.0212	0.0172	0.0144	0.0116
E		3.5598	3.5515	3.5415	3.5296	3.5253	3.5170	3.5131	3.5131	3.5025	3.4948	3.4862

**Table 7:** The highest forecast price of options under different constraint satisfactory degrees.

	β	0.0	0.1	0.2	0.3	0.4	0.5	0.6	0.7	0.8	0.9	1.0
*K*												
3.425		0.0368	0.0363	0.0378	0.038	0.0372	0.0352	0.0300	0.0196	0.0204	0.0218	0.0240
3.450		0.0436	0.0431	0.0448	0.0485	0.0442	0.0423	0.0372	0.0281	0.0294	0.0314	0.0345
3.500		0.0591	0.0587	0.0608	0.0601	0.0607	0.0601	0.0569	0.0507	0.0534	0.0570	0.0616
3.550		0.0777	0.0779	0.0804	0.0801	0.0820	0.0840	0.0834	0.0787	0.0834	0.0882	0.0942
3.575		0.0885	0.0893	0.0921	0.0914	0.0953	0.0983	0.0986	0.0944	0.1001	0.1054	0.1118
3.600		0.1006	0.1021	0.1054	0.1058	0.1106	0.1144	0.1151	0.1114	0.1182	0.1236	0.1307
3.625		0.1141	0.1164	0.1205	0.1127	0.1278	0.1322	0.1331	0.1299	0.1376	0.1433	0.1509
3.650		0.1290	0.1321	0.1371	0.1411	0.1462	0.1510	0.1523	0.1495	0.1580	0.1641	0.1721
3.675		0.1451	0.1488	0.1550	0.1552	0.1654	0.1706	0.1724	0.1702	0.1794	0.1859	0.1942
3.700		0.1619	0.1664	0.1737	0.1744	0.1853	0.1911	0.1933	0.1918	0.2017	0.2085	0.2170
RMSE		0.0096	0.0109	0.0145	0.0149	0.0200	0.0232	0.0236	0.0212	0.0276	0.0324	0.0387
E		3.5598	3.5515	3.5415	3.5296	3.5253	3.5170	3.5131	3.5131	3.5025	3.4948	3.4862
